# Evaluating scaling models in biology using hierarchical Bayesian approaches

**DOI:** 10.1111/j.1461-0248.2009.01316.x

**Published:** 2009-07

**Authors:** Charles A Price, Kiona Ogle, Ethan P White, Joshua S Weitz

**Affiliations:** 1 School of Biology, Georgia Institute of TechnologyAtlanta, GA 30332, USA; 2 Departments of Botany and Statistics, University of Wyoming, LaramieWY 82071, USA; 3 Department of Biology and the Ecology Center, Utah State UniversityLogan, UT 84322, USA; 4 School of Physics, Georgia Institute of TechnologyAtlanta, GA 30332, USA

**Keywords:** Allometry, elastic similarity, fractal, geometric similarity, hierarchical Bayes, leaves, scaling, stress similarity, trees

## Abstract

Theoretical models for allometric relationships between organismal form and function are typically tested by comparing a single predicted relationship with empirical data. Several prominent models, however, predict more than one allometric relationship, and comparisons among alternative models have not taken this into account. Here we evaluate several different scaling models of plant morphology within a hierarchical Bayesian framework that simultaneously fits multiple scaling relationships to three large allometric datasets. The scaling models include: inflexible universal models derived from biophysical assumptions (e.g. elastic similarity or fractal networks), a flexible variation of a fractal network model, and a highly flexible model constrained only by basic algebraic relationships. We demonstrate that variation in intraspecific allometric scaling exponents is inconsistent with the universal models, and that more flexible approaches that allow for biological variability at the species level outperform universal models, even when accounting for relative increases in model complexity.

## Introduction

The past several decades have seen a resurgence of interest in the field of biological scaling. The publication of several compendia of allometric relationships for animals (Peters 1983; Calder 1984) and plants (Niklas 1994) have highlighted what appear to be recurrent scaling patterns within and across taxa. Examples of allometric relationships that address organismal form and function include: relationships between morphological traits, such as tree diameter and tree height (McMahon & Kronauer 1976; Niklas & Spatz 2004), or relationships between organism size and physiology, such as body mass and metabolic rate (Kleiber 1932; Heusner 1982; White & Seymour 2003; Savage *et al.* 2004).

The existence of such recurrent scaling patterns has motivated attempts to model the scaling of biological phenomena based on physical first principles. In the case of plants, several scaling models have garnered significant attention due to their proposed generality and because they yield multiple, testable predictions (Table 1). These include the biomechanical models for the scaling of ‘life’s dimensions’ first introduced by McMahon (1973) and McMahon & Kronauer (1976) and more recent efforts invoking fractal branching networks (West *et al.* 1997, 1999; Price & Enquist 2007; Price *et al.* 2007). Understanding how well these models characterize allometric scaling behaviour provides important insights into the processes underlying observed allometries and the level of model complexity necessary for addressing particular biological scaling questions.

**Table 1:** Categorization of scaling exponents for six different scaling models of allometric relationships among plant properties. Every element of the table denotes an exponent, where *r* is basal stem or petiole radius, *l* is the plant height or leaf length, *A* is the surface area of an individual or of the leaves of a plant, and *M* is the plant or leaf mass. The top row represents the independent variable, e.g. the two-thirds in the upper left cell denote that under elastic similarity *l*∝ *r*^2/3^. The top four models predict universal scaling exponents whereas the bottom two predict variable exponents that are not restricted to particular numerical values

Model (category)	*r*	*l*	*M*
Elastic similarity (universal)			
*r*	–	–	–
*l*	2/3	–	–
*M*	8/3	4	–
*A*	NA	NA	NA
Stress similarity (universal)			
*r*	–	–	–
*l*	1/2	–	–
*M*	5/2	5	–
*A*	NA	NA	NA
Geometric similarity (universal)			
*r*	–	–	–
*l*	1	–	–
*M*	3	3	–
*A*	2	2	2/3
WBE (universal)			
*r*	–	–	–
*l*	2/3	–	–
*M*	8/3	4	–
*A*	2	3	3/4
PES (constrained)			
*r*	–	–	–
*l*	*b*/*a*	–	–
*M*	(2*a* + *b*)/*a*	(2*a* + *b*)/*b*	–
*A*	1/*a*	1/*b*	1/(2*a* + *b*)
SPAM (specialized)			
*r*	–	–	–
*l*	*η*	–	–
*M*	*ϕ*	*ϕ*/*η*	–
*A*	*λ*	*λ*/*η*	*λ*/*ϕ*

Dashes denote the symmetric or isometric elements. NA indicates that the model does not make specific predictions for the corresponding scaling exponent.

WBE, model of West *et al.*; PES, model of Price *et al.*; SPAM, specialized allometry model.

Empirical tests of these scaling models typically rely on traditional approaches that fit simple linear regressions to bivariate plots of log-transformed data for a single predicted relationship (i.e. for one particular property vs. another). The confidence intervals for key parameters (e.g. slopes) are examined to determine whether or not they contain a particular scaling model’s predicted value. This approach ignores the fact that many allometric models make predictions for a suite of interconnected relationships among multiple properties and does not allow for exploration of varying degrees of model complexity. Another issue is that classical methods for estimating the coefficients describing how a particular property of an organism scales with another property either ignore uncertainty in one of the variables (e.g. the ‘*x*-variable’) or employ relatively restrictive assumptions about variance terms when accounting for uncertainty in both variables (Warton *et al.* 2006). To address these issues, we describe a hierarchical Bayesian (HB) approach that simultaneously evaluates multiple predicted scaling relationships and explicitly accounts for uncertainty in all measured traits. This approach is applied to compare intraspecific differences in allometric relationships of plant morphology based on whole-plant and leaf datasets.

The allometric models we considered can be divided into three major categories: universal, constrained, and specialized (Table 1). Universal models are derived from physical first principles and are expected to be universally applicable both within and across species. These models yield specific numerical predictions for a suite of allometric exponents, and the numerical values are assumed to be the same across all individuals and species. In constrained models, the scaling exponents may take on a wide array of numerical values, but these values are ‘constrained’ by physical design principles. That is, assumptions about biological limitations result in the scaling exponents for one allometry to be expressed as a function of the exponents describing other allometries. In contrast, specialized models are highly flexible ones that do not arise from underlying physical or biological assumptions. In these models, the allometric exponents are only constrained by simple logical (i.e. algebraic) relationships such that each species may take-on unique (or ‘specialized’) exponent values. Our objective is to compare the predictive power of different scaling models, representing different levels of complexity, while accounting for the fact that universal models inherently involve fewer free parameters than constrained models, which involve fewer free parameters than specialized models. We utilize three large allometric datasets of plant and leaf traits containing in total 2362 individuals from 110 species to evaluate the ability of the universal, constrained, and specialized models to fit observed data and to determine if the universal models satisfactorily capture observed allometric patterns.

We first define the scaling models to be compared and highlight the predictions made by each model. Next, we describe an HB approach for evaluating the predictive power of scaling models of varying complexity. We compare the performance of the different scaling models in two primary ways: (i) we compare the posterior distributions of the population-level scaling exponents to predictions from universal models, and (ii) we rigorously evaluate the ability of each scaling model to predict the observed data via model goodness-of-fit comparisons and estimates of posterior predictive loss.

## Scaling models

Allometric scaling models often make multiple predictions about how aspects of organismal form or function vary with some measure of size (e.g. length or mass). The models considered here have all been applied to the study of plant traits. However, both the theoretical models we test and the HB framework we employ are more general and could be applied to other taxa. To begin, consider the relationships between plant or leaf mass (*M*), whole-plant or individual leaf surface area (*A*), plant height or leaf length (*l*), and basal stem or petiole radius (*r*). Given these traits, we may be interested in any of the six possible scaling relationships, e.g. between *r* and *l* or between *M* and *A* and so on. If power-law scaling is observed, only three of the six relationships are independent. In a universal model, the three independent scaling exponents take-on particular numerical values that are applicable to all species. In a specialized scaling model, each of the three scaling exponents is free to vary at the species-level without any constraints. Finally, constrained models represent an intermediate complexity where some, but not all, of the exponents are constrained relative to each other due to hypothesized biological limitations. Table1 lists the examples of each type of scaling model.

Next, we outline the three groupings of scaling models: four universal models, one constrained model, and one specialized model. All six are classified as power-law models, which predict relationships of the form log(*y*) = log (α) + β log(*x*), where α is the normalizing constant and β is the scaling exponent.

### Universal models

Examples of models that predict universal scaling exponents of plant form and function are stress similarity (STRESS; McMahon & Kronauer 1976), elastic similarity (ELASTIC; McMahon & Kronauer 1976), geometric similarity (GEOM; Rubner 1883; Niklas 1994), and the fractal branching model of West *et al.* (1999), hereafter WBE. In each model, some physical optimization principle is invoked to explain the origin of allometric exponents, and no free parameters are needed in terms of species-specific scaling other than the normalizing constants.

#### Stress and elastic similarity

STRESS assumes that a constant maximum biomechanical stress level is maintained throughout the branches of the trees. Similarly, ELASTIC assumes that the ratio of a branch’s deflection to its length remains constant across branches of different sizes (McMahon & Kronauer 1976). Both models are derived from biophysical principles and yield primary (biomechanical similarity, a testable assumption) and ancillary (particular scaling exponents, Table 1) predictions. These two models make different predictions for the scaling exponents relating length, radius, and mass, but neither makes explicit predictions for how total leaf area should scale with other plant traits.

#### Geometric similarity

The biological application of this model (GEOM) was first proposed by Galileo as a means for predicting the scaling of animal limb bone dimensions (Calder 1984). Other applications include the scaling of energy use in dogs (Rubner 1883). GEOM assumes that length and radius scale isometrically with each other. We treat GEOM as a null model for scaling in plants without regard to the functional arguments upon which it is based (Niklas 1994).

#### Fractal branching network

The fractal branching model (WBE) assumes that internal resource delivery networks have been selected to minimize resistance to flow (West *et al.* 1997, 1999). WBE assumes that the structural components of plants (i.e. branches) are elastically similar, thus for the scaling of plant dimensions (height, stem diameter) with mass, the model makes identical predictions to that of McMahon’s elastic similarity. However, WBE also provides predictions about the scaling of surface area and dynamic aspects of organismal metabolism (West *et al.* 1999). Thus, within our analysis, the extended applicability of the WBE model is reflected in the greater number of predicted scaling exponents compared with the elastic and stress similarity models (see Table 1).

### Constrained models

Models with constrained exponents are those that invoke biological mechanisms to constrain the scaling exponents relative to each other. This implies that values for the scaling exponents cannot be established *a priori*, but relationships among them can.

*PES*: Price *et al.* (2007) provide an example of a constrained exponent model, which is referred to as PES. In PES, the overall design is a fractal branching network with the same underlying mathematical structure as WBE. The PES model differs from WBE because it does not assume a single optimal exponent. Instead, PES allows the branch-length and branch-radii relationships to vary between species. This results in a set of relations that requires only two, potentially species-specific, scaling parameters (*a* and *b*) to be estimated from data. All other predicted exponents are explicit functions of *a* and *b* (Table 1).

### Specialized model

A specialized allometry model (SPAM) is one in which all independent scaling exponents are free to vary, i.e. there are no constraints among the three independent exponents (*η*, *ϕ*, and *λ*; Table 1). The only assumption underlying the SPAM model is that the relationships between the variables are power laws and as such this is a purely empirical model. Thus, knowing any three of the scaling relationships allows one to determine the other three through algebraic manipulation of the power law equations.

## Methods

### Data sources

Three data sources were utilized in this study; these sources were selected because they included observations for multiple species for at least three of the four variables considered here (*l*, *r*, *A*, *M*). The first describes the ‘average’ properties of whole trees and is from the Cannell (1982) data compendium. Cannell reports stand-level mean tree height (*l*, m), stem biomass (*M*, kg), leaf mass, and stem diameter (2*r*, cm) for multiple, even-aged stands, providing observations for 256 stands representing 14 species (Supporting Information, Appendix S1, Table S1). To conform to the predictions of the models invoking fractal similarity (WBE and PES), we assumed isometric relationships between whole-tree leaf surface area (*A*, cm^2^) and leaf mass, and whole-tree and stem biomass (Price *et al.* 2007).

The second dataset is for plants from the Sonoran Desert (Price *et al.* 2007). The dataset contains measurements of plant height (*l*, m), basal stem diameter (2*r*, cm), and plant mass (*M*, kg) for 1180 individual plants representing 49 species (Table S2).

The third dataset contains observations for leaves representing 926 individual leaves from 47 species (Table S3). Data were collected during the summer of 2007 from trees in the greater Atlanta region (Lat/Long 33°75′, −84°38′). The species were selected for collection based on local availability. For each fresh leaf, major axis length (*l*, mm) and petiole diameter (2*r*, mm*,* average of minor and major axes) were measured with digital calipers. Fresh leaves were digitally scanned and surface area (*A*, mm^2^) was measured with image analysis software (Scion Image Beta 4.0.2; http://www.scioncorp.com). All leaves were dried in a drying oven until a constant dry mass (*M*, g) was attained. These data were collected for as large a range of leaf sizes as could be found for each species.

### HB model

We chose to implement a HB framework (Ogle & Barber 2008) to simultaneously fit the scaling models to each dataset for four primary reasons. First, it can easily accommodate a multivariate likelihood that quantifies correlations between different traits in addition to accounting for variation explained by the scaling model(s). Second, we essentially treat *r* as the ‘independent’ variable and explicitly account for measurement errors in *r*. Third, for scaling models that allow for species-specific exponents, we specify a hierarchical parameter model that allows under-represented species (i.e. those with few observations) to ‘borrow strength’ from well-represented species. Fourth, the HB framework is based on a conditional probability model that describes uncertainty in all stochastic components (e.g. data and parameters) and quantifies relationships between these components (Ogle & Barber 2008). This framework yields the joint posterior distribution for all unknown quantities, conditional on the data and the model structure, and inferences based on the posterior are very straightforward (Carlin *et al.* 2006; Ogle & Barber 2008). Next, we highlight the important elements of the HB model that we implemented (see Appendix S2 for a detailed explanation of the models and implementation procedures).

For observation *i* (*i*=1, 2, 3, …, *N*_*k*_ for dataset *k*), we employ a Berkson error-in-variables model (Dellaportas & Stephens 1995) to account for measurement errors in *r*_*i*_, which we assume are log-normally distributed: 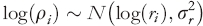
(1) where *ρ*_*i*_ is the ‘true’ or latent radius and 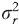
is the measurement error variance. For a given dataset, all scaling models use the same *ρ* values. On the log-scale, the multivariate normal likelihood for vector *i* containing the other observed traits is: 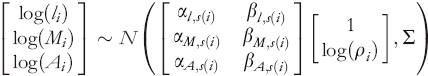
(2) where the *α*s are the normalizing constants and the *β*s are the scaling exponents for the relationships between *l*, *M*, or *A* and *ρ*, Σ is a 3 × 3 covariance matrix, and *s*(*i*) indicates ‘species *s* associated with observation *i*’. We employ a hierarchical prior that models species-specific parameters as coming from an overall (or ‘global’) population that is defined by population-level parameters (e.g. Clark *et al.* 2005; Ogle & Barber 2008). For variable *Y* (*Y*= *l*, *A*, or *M*) and species *s*: 
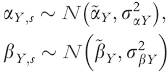
(3) where 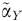 and 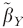 are the global normalizing constants and scaling exponents, respectively, and 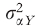 and 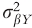 are the variances that describe variability between species with respect to these parameters. Equations 2 and 3 represent the most flexible model (SPAM) where *β*_*l*_, *β*_*M*_, and *β*_*A*_ are equivalent to *η*, *ϕ*, and *λ*, respectively in Table 1. For all scaling models, we allow the αs to differ between species. However, we may adjust the model for the *β*s such that, for the universal models, we drop the *s* subscripts and assume particular values for the *β*s (Table 1). For PES, we apply eqn 3 to *β*_*l*_ and *β*_*A*_, and based on predictions in Table 1 involving parameters *a* and *b*, *β*_*M*_ = *β*_*l*_ + 2, *a*=1/*β*_*A*_, and *b* = *β*_*l*_/*β*_*A*_. We chose a relatively informative prior for 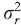
(eqn 1) and assigned non-informative priors to all remaining parameters. We used Markov chain Monte Carlo methods to approximate the joint posterior distribution associated with this likelihood and parameter models. We implemented the models in WinBUGS (Lunn *et al.* 2000), a general-purpose statistical software package for conducting Bayesian analyses (code provided in Appendix S3).

## Results

### Assessing universality of allometric scaling exponents

**Figure 1:**
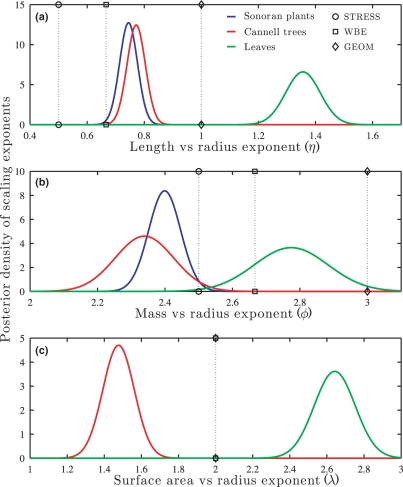
Posterior distributions for the global exponents in the specialized allometry model (SPAM). The dashed vertical lines represent exponent values predicted by the universal models (Table 1). None of the universal models enjoys strong support across all allometries or all datasets. Bayesian credible intervals (BCI) and the exponent predictions from the universal models are reported in Table 2. Note that the elastic similarity model makes the same predictions as the model of West *et al*. (1999) for the scaling of mass and length. In addition, stress and elastic similarity models do not make predictions for the scaling of surface area.

We evaluated the posterior distributions for the population-level (or global) and species-specific exponents obtained under the SPAM described by eqns 1–3. We compared the 95% Bayesian credible intervals (BCI) for the global exponents (the 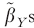 in eqn 3) in the SPAM model with those predicted by each of the universal scaling models (Fig. 1 and Table 2). None of the 95% BCI contained the predicted exponent values of the GEOM model, but the 95% BCI did include the predicted values in three out of twenty analyses for the WBE, ELASTIC, and STRESS models (Table 2). Specifically, the 95% BCI for the global scaling exponents obtained for the *M* vs. *r* relationship for the Cannell data contained the exponent predicted by the STRESS model. In addition, the posterior distribution for the *M* vs. *r* scaling exponent for the leaf data overlaps the values predicted by the WBE and ELASTIC models (Fig. 1). To investigate whether the choice of independent variable influenced our findings we repeated the HB analyses using *M* as the independent variable (Fig. S1). For brevity, we do not report the full results here, but a similar story emerges: no universal model performs well across all relationships and datasets.

**Table 2:** Posterior mean, SD, and 95% Bayesian credible interval (BCI) limits based on the lower 2.5th percentile (2.5%) and the upper 97.5th percentile (97.5%) for the global scaling exponents associated with the most flexible model, i.e. specialized allometry model or SPAM (see Fig. 1). The predicted numerical values for the exponents in the universal models are in the middle four data columns (‘Model predictions’). Shaded gray cells indicate predicted values that were contained in the 95% BCI for the SPAM model. The rightmost four columns (‘Per cent contained’) contain the percentage of species-level exponent BCI that contained a given model’s predicted exponent value. For example, for the *l* vs. *r* relationship within the Cannell dataset, 14.3% of the species-level BCI included the WBE model’s predicted exponent value (or equivalently the elastic model)

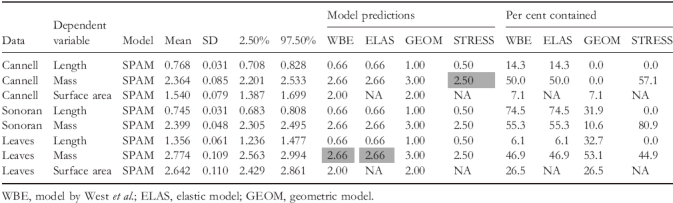

**Figure 2:**
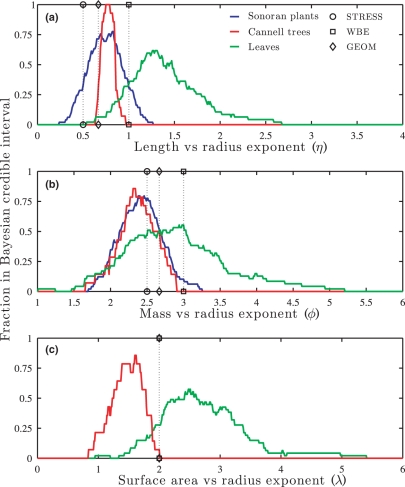
Smoothed frequency histograms for the fraction of the Bayesian credible intervals (BCI) for each species-specific scaling exponent that include the exponent value indicated on the *x*-axis. The predicted exponent values from the universal models are plotted for reference (horizontal dashed lines). Note that the stress and elastic similarity models do not make predictions for the scaling of surface area.

To explore the variability of the species-specific scaling exponents for each relationship (i.e. *l* vs. *r*, *M* vs. *r*, *A* vs. *r*), we tallied the number of species-specific 95% BCI from the SPAM model that contained any particular exponent value (Fig. 2). We did not find a single case where a universal scaling prediction was contained in the 95% BCI for all species-specific exponents. Moreover, none of the universal scaling models was consistent with all of the allometric relationships in these datasets. For example, across all datasets, less than 50% of the length–radius exponents’ BCI contained the predicted values given by the STRESS, WBE, or GEOM models (Fig. 2a). The highest fraction was observed for STRESS with the Sonoran plant dataset, for which *c.* 75% of species-specific mass–radius exponents’ BCI contained the predicted STRESS values (Fig. 2b). Detailed results for all three datasets are reported in Tables S1–S3.

### Comparing the predictive power of models of varying complexity

**Figure 3:**
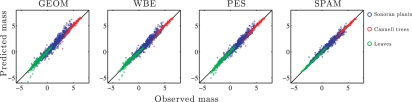
Illustration of the improvement in predictive power with more flexible scaling models. The predicted mass values are the posterior means for replicated data. The black line in each figure is the 1 : 1 line. Note that the model of Price *et al*. (2007); PES) and the specialized allometry model (SPAM) have less scatter about the 1 : 1 line compared with the universal models, WBE model of West et al. (1999) and the geometric model (GEOM).

We conducted two sets of analyses to compare how well models of varying complexity captured the observed data. First, we used eqn 1 to generate replicated data for each dependent variable (Gelman *et al.* 2004), yielding posterior predictive distributions for each observation in each dataset, for each model. If a given model perfectly predicted the data, all points would lie exactly on the 1 : 1 line in an observed vs predicted plot. In general, the models fit the data very well as the points were tightly clustered around the 1 : 1 lines (Fig. 3), but clustering around the 1 : 1 line was higher for the SPAM model compared with the other models. The greatest deviations occurred for the universal models, and this was especially pronounced for the leaf data. These goodness-of-fit differences are also reflected in the variance (or SD) estimates for *l*, *M*, and *A*. That is, the covariance matrix Σ in eqn 2 describes the residual variability after having accounted for variation in *l*, *M*, and *A* explained by the scaling models. Across all datasets and traits, the residual variance was always smallest for the SPAM model (Table S4).

Since more complex (i.e. parameter-rich) models are expected to outperform simpler models in terms of goodness-of-fit, we also computed the posterior predictive loss (*D*), which penalizes for model complexity (Gelfand & Ghosh 1998). *D* was always lowest for SPAM, typically followed by PES, and the universal models generally had the highest *D* values (Table 3). The rankings of the universal models, with respect to *D*, varied depending on the dataset and trait of interest. For nearly all dataset–trait combinations, *D* was significantly lower for SPAM compared with the universal models. The one exception occurred for length (*l*) in the Cannell dataset, where *D* was lowest for SPAM, but it was not significantly different from the *D* obtained for ELASTIC and WBE. In many cases, *D* was also significantly lower for SPAM compared with WBE, but there are instances in which the smaller *D* values for SPAM were contained in the 95% BCI for the associated WBE’s *D* value (i.e. length for all datasets). Overall, comparisons of *D* between models within each dataset indicate strong support for species-specific exponents as represented by SPAM, moderate support for PES, and providing comparatively little support for the universal scaling models.

**Table 3:** Posterior predictive loss (*D*; mean) and its 95% Bayesian credible interval (BCI; lower 2.5th and upper 97.5th percentiles) for the six models for length, mass, area, length and mass together, and all three traits combined. Lower values of *D* indicate greater support for the corresponding model; model-trait *D*-values may be considered different if the 95% BCI for one model–trait combination does not contain the posterior mean for another model–trait’s *D* (only applicable to comparisons within a given trait category)

		Cannell			Sonoran			Leaves		
Model	Trait	Mean	2.50%	97.50%	Mean	2.50%	97.50%	Mean	2.50%	97.50%
ELASTIC	Length	5.369	4.369	6.511	91.28	83.82	99.37	45.47	41.03	50.12
STRESS	Length	7.522	6.179	9.041	107.6	98.74	117	61.61	55.77	67.63
GEOMETRIC	Length	6.78	5.558	8.191	116.2	106.9	126.2	25.12	22.39	28.14
WBE	Length	5.374	4.395	6.503	91.32	84.2	99.31	45.54	41.19	50.25
PES	Length	5.791	4.633	7.158	75.69	69.29	82.41	9.989	8.369	11.9
SPAM	Length	4.902	3.982	5.957	75.6	69.07	82.59	8.567	7.111	10.26
ELASTIC	Mass	16.61	13.77	19.87	273.2	249.4	297.6	60.57	52.66	68.96
STRESS	Mass	13.23	10.91	15.85	245.6	223.8	267.4	58.92	51.3	67.13
GEOMETRIC	Mass	29.04	24.15	34.76	386.2	354.5	420.2	75.71	66.36	85.76
WBE	Mass	16.59	13.72	19.87	273.4	249.9	298	60.91	53.19	69.22
PES	Mass	14.77	11.86	18.11	260	235.4	285.5	60.01	50.36	70.68
SPAM	Mass	7.464	6.014	9.132	204.6	185.7	224.2	18.8	14.95	23.29
GEOMETRIC	Area	21.6	17.87	25.84	NA	NA	NA	72.62	64.35	81.76
WBE	Area	21.62	17.91	25.8	NA	NA	NA	72.71	64.35	81.75
PES	Area	13.61	10.42	17.33	NA	NA	NA	22.62	17.49	28.67
SPAM	Area	9.6	7.734	11.8	NA	NA	NA	19.36	15.19	24.19
ELASTIC	Length and mass	21.98	18.91	25.44	364.5	337.6	391.6	106	96.22	116.3
STRESS	Length and mass	20.76	18.12	23.73	353.2	328	378.5	120.5	109.9	131.6
GEOMETRIC	Length and mass	35.82	30.52	41.95	502.4	465.2	542.3	100.8	90.39	111.8
WBE	Length and mass	21.97	18.93	25.46	364.7	338.2	392.3	106.5	96.64	116.8
PES	Length and mass	20.56	17.6	23.88	335.7	309.1	363.3	70	60.42	80.69
SPAM	Length and mass	12.37	10.6	14.32	280.2	257.9	303	27.37	22.52	33
GEOMETRIC	Length, mass, and area	57.43	49.54	66.29	NA	NA	NA	173.5	157.1	191.3
WBE	Length, mass, and area	43.59	37.77	50.02	NA	NA	NA	179.2	162.3	197.3
PES	Length, mass, and area	34.18	29.39	39.58	NA	NA	NA	92.62	79.14	108.1
SPAM	Length, mass, and area	21.97	19.2	25.15	NA	NA	NA	46.73	38.24	56.78

WBE, model of West *et al.*; PES, model of Price *et al.*; SPAM, specialized allometry model; NA, not applicable.

Finally, the estimates of the trait correlation coefficients that describe the off-diagonals of the covariance matrix Σ in eqn 2 indicate the importance of simultaneously considering all traits within a multivariate modelling framework. These correlation coefficients describe the residual correlation between pairs of traits after accounting for variation explained by the scaling models. Of the 34 possible coefficients, 29 were significantly different than zero, indicating the existence of strong residual trait correlations (Table S4). Posterior estimates for the components of Σ and for σ_*r*_^2^ are given in Table S4.

## Discussion

The typical approach to evaluating models for allometric scaling relationships is to compare a single prediction from a single allometric model to data and determine whether or not the model is consistent with the data (White & Seymour 2003; Bokma 2004; Glazier 2006). Several such studies to date have indicated significant variability in both intraspecific and interspecific allometric scaling patterns (Bokma 2004; Glazier 2006; Muller-Landau *et al.* 2006a). Even with multiple scaling relationships, each is typically analysed in isolation, so most analyses are equivalent to single relationship comparisons presented together in the same study (Savage *et al.* 2004; Anfodillo *et al.* 2006; Muller-Landau *et al.* 2006b). Evaluating a scaling model based on a single prediction has two major limitations. First, it ignores the fact that most allometric models make predictions for a suite of relationships. As a result, comparisons of singular relationships ignore one of the strengths of these synthetic theories and may therefore be biased towards rejecting universal models or may provide reduced power for distinguishing among models. Second, single scaling predictions have been used to evaluate mechanistic scaling models against null models that do not fit data well and that do not offer a meaningful comparison in terms of competing biological theory. Thus, when comparing different models with one another it can often be difficult to reject either model (if they make similar predictions) based on simple regression analyses, making it difficult to draw inferences about the underlying biological processes.

The approach we have presented here differs from traditional approaches to fitting and evaluating scaling models in that this is only the second study that we are aware of to examine multiple predictions simultaneously (Dietze *et al.* 2008). Moreover, this study presents the first rigorous intermodel comparison of multiple scaling models. We also expand the breadth of taxonomic and functional groups explored compared with the previous work (Dietze *et al.* 2008), including 2362 individuals from 110 species. These species represent a broad array of phylogenetic, morphological, functional, and life history groups including: angiosperms and gymnosperms; annuals and perennials; monocots and dicots; C3, C4, and CAM (crassulacean acid metabolism) photosynthetic pathways; and herbaceous, succulent, and woody species. The HB framework that we employed was able to accommodate this diversity by allowing each species to potentially be described by a species-specific allometry that can be thought of as arising from a global ‘plant’ allometry. In addition, this approach allows the explicit incorporation of important sources of variability that are typically ignored. Finally, we utilize a number of different model comparison criteria, providing a more complete evaluation than simply evaluating confidence intervals for slope estimates obtained from regression analyses that do not explicitly incorporate multiple sources of uncertainty.

Our analysis shows that the maximally flexible empirical models provide better fits to the data than the comparatively restrictive mechanistic models, even after considering differences in the number of parameters, or model complexity. The posterior intervals for the global exponents from the SPAM model did not consistently contain theoretical predictions for any of the universal models. In one case, the predicted scaling exponent for a single relationship was well supported by the data (*M* vs. *r* in leaves was consistent with the WBE and ELASTIC models), but the predictions of these two models for the other two scaling relationships failed to describe the overall pattern in the data (Fig. 1). As such, the scaling behaviour of the four plant properties considered here were not captured by any of the universal models that we evaluated in any of the our datasets. However, when only considering plants, the posterior distributions for the *l* vs. *r* and *M* vs. *r* scaling exponents strongly overlap for the Cannell and Sonoran datasets (Fig. 1). This agreement occurred despite the fact that these datasets differ significantly in their collection methods, taxonomic coverage, and functional group composition. The strong overlap in their global distributions suggests that there may exist a tendency towards a particular scaling allometry that applies across species, but this ‘global’ allometry differs from those predicted by existing scaling theories.

The BCI for the species-specific scaling exponents also suggest that no universal model is supported consistently across species, allometric relationships, and datasets. Some models enjoy support for particular combinations of dataset and allometric relationships. For example, the WBE and ELASTIC models perform well for Sonoran species, with 75% of the credible intervals for the *l* vs. *r* relationship and 55% of the credible intervals for the *M* vs. *r* relationship containing the WBE and ELASTIC model predictions. The greater agreement at the species vs. the global levels occurred because the posterior intervals for the species-specific exponents were broader, spanning a wider range of values. Species-level estimates based on the Cannell data somewhat agree with the STRESS, WBE, and ELASTIC exponents for the *M* vs. *r* relationship, but they generally do not agree with the *l* vs. *r* and *A* vs. *r* scaling relationships predicted by these models. Moreover, the posterior distributions for the scaling exponents varied greatly across species, datasets, and allometric relationships; thus, any model that predicts a single universal exponent will not explain this variability.

As expected, the more flexible models (e.g. PES and SPAM) explained more variation in the observed plant data than the less flexible models (e.g. WBE, GEOM, ELASTIC, and STRESS). The universal models we considered did perform reasonably well in predicting the scaling of plant form when looking at the data for all taxa combined (Fig. 3); however, some systematic error was produced by each of these models. For example, for a given radius, both the GEOM and WBE models tend to overpredict mass at large sizes, particularly among the Sonoran Desert species. Similarly, PES tends to underpredict mass for leaves at small sizes. Thus, caution should be used when assuming universal exponents in ecological studies.

Of the mechanistic models we explored, PES consistently outperformed the universal models. This improved fit could result simply from the increase in model parameters. However, as seen in Table 3, despite penalizing for model complexity, the posterior predictive loss for PES was consistently lower than for any of the universal models. This suggests that the PES model performs better because it allows for variability in network or morphological design that is more consistent with the growth and architecture of real plants. This also highlights the need to test model assumptions in addition to model predictions; in this example, the underlying assumptions could be evaluated by directly testing the scaling of vascular elements (e.g. McCulloh *et al.* 2003; Anfodillo *et al.* 2006; Weitz *et al.* 2006; Mencuccini & Holtta 2007).

Although we present several summary statistics in our analysis, we caution against over-reliance on any one metric. By considering all of the statistics and patterns evaluated here, a consistent story emerges: more flexible models perform better than those with fixed parameters. This improved performance appears to be robust to increases in the number of fitted parameters, suggesting that intraspecific allometric modelling efforts would benefit by explicitly acknowledging important sources of variability between species. Differences between mechanistic universal models and species-specific empirical models of plant growth and form may be addressed by incorporating additional influences on scaling relationships (Muller-Landau *et al.* 2006a), addressing potential departures from power law behaviour (Savage *et al.* 2008), or grouping plants into functional groups that are under similar constraints and therefore share similar allometric relationships. While the assumption of universal allometric behaviour may be a useful first approximation for some broad-scale comparisons, accounting for the variability observed in these biologically relevant phenomena will ultimately lead to more realistic models of plant form and function.

It should be noted that our application of some of these models to leaves (in particular, WBE) extends beyond their intended scope. However, extensions of WBE have successfully predicted the scaling of leaf morphology (Price & Enquist 2007). Thus, it is within the context of comparing the original WBE model with a subsequent extension (PES) that we include the WBE model in our analysis of the leaf data. Moreover, the mechanistic arguments underlying the other universal models (STRESS, ELASTIC, or GEOM) apply to leaves in principle. Additionally, the predictions from WBE that we use are only strictly valid in the limit of a large number of branching generations (Enquist *et al.* 2007; Savage *et al.* 2008). This would be consistent with the fact that WBE performs best for trees in the Cannell dataset. Finally, and perhaps most importantly, all of our analyses are of intraspecific allometric relationships in plants. While these certainly provide valid tests of the universal plant models, our results do not apply directly to other types of interspecific scaling relationships, such as the scaling of adult metabolic rate in determinately growing mammals (Kleiber 1932).

We also note that the HB framework has a number of benefits for analyses of allometric scaling. First, it allows the explicit incorporation of uncertainty in both dependent and independent variables. Second, as noted before, it facilitates estimation of multiple allometric exponents and normalizing constants within a unified statistical framework. Third, it allows direct linkages between multiple traits across multiple species, thus accounting for correlations between traits that are not completely explained by the scaling models. Finally, this approach allows the simultaneous fitting of all allometric scaling models, enabling a rigorous evaluation of the different scaling models via comparisons of multiple model fit indices.

In sum, there is little support for any of the universal scaling models as descriptions of plant morphology at the intraspecific level. Estimated allometric exponents exhibit a fairly broad range, and while all of the scaling patterns that we analysed do exhibit some degree of central tendency, this is not adequately captured by any one of the universal scaling models. As such, our analyses suggest that scaling models could benefit by attempting to incorporate more complexity in order to more accurately capture biological variability. Determining the principal axes of variation governing the scaling of plant form will be important for these efforts. Finally, we have demonstrated that a HB framework is well suited for performing analyses of this type due to its inherent flexibility, hierarchical structure, and explicit integration of multiple levels of variability.

## SUPPORTING INFORMATION

**Figure S1** Posterior distributions for the global exponents in the specialized allometry model for mass as the independent variable.

Tables S1–S3 Intraspecific exponent estimates for the Cannell, Sonoran, and leaf datasets from the specialized allometry model.

**Table S4** Posterior means for terms associated with the covariance matrix Σ and the radius measurement error variance (σ_*r*_).

**Appendix S1** Intraspecific exponent estimates.

Appendix S2 Hierarchical Bayesian model and results.

Appendix S3 WinBUGS code.
