# Supplementary material for: Evaluating scaling models in biology using hierarchical Bayesian approaches
Source: Ecol Lett. 2009 Jul;12(7):641–51. doi: 10.1111/j.1461-0248.2009.01316.x (PMC2730548; doi:10.1111/j.1461-0248.2009.01316.x)
Supplement: Appendix S2 — Hierarchical Bayesian model and results. [file ele0012-0641-SD1.doc]

**SUPPORTING INFORMATION**

**Appendix S2** *Hierarchical Bayes model and results*

**Methods: Hierarchical Bayesian model**

We employed a hierarchical Bayesian (HB) framework to simultaneously fit the different scaling models to each dataset (Dietze et al. 2008). We choose this method for four primary reasons. First, the HB framework can easily accommodate a multivariate likelihood that explicitly quantifies correlations between observations of different plant characteristics (e.g., *l*, *r*, *A*, *M*) in addition to accounting for variation explained by a particular scaling model (e.g., Ogle & Barber 2008). Second, although we essentially treat *r* as the “independent” variable, we explicitly account for observation errors in *r* via a Berkson type error-in-variables model (Dellaportas & Stephens 1995). As noted by Dellaportas and Stephens (1995), the Bayesian approach to accounting for errors in the “independent” variable is preferred over classical approaches such as reduced major axis (RMA) regression because we are “able to make inferences under a truly representative model specification;” conversely, classical approaches employ restrictive assumptions about the variances terms and often encounter problems with numerical stability in non-linear models. Third, for scaling models that allow for species-specific exponents, a hierarchical parameter model for the exponents allows under-represented species (i.e., those with few observations) to “borrow strength” from well-represented species. That is, the exponent values for under-represented species will be partly informed by the values predicted for well-represented species. Fourth, the HB framework treats all observations and unknown quantities (e.g., scaling exponents, intercepts, (co)variance terms, latent variables) as stochastic variables, and a conditional probability model describes uncertainty in these components and how they are related to each other (Ogle & Barber 2008). This framework yields the joint posterior probability distribution for all unknown quantities, conditional on the data and the model structure, and inferences based on the posterior are very straightforward (Carl*in et a*l. 2006; Ogle & Barber 2008). For example, we can directly compare the posteriors for the scaling exponents to those predicted by the different scaling models to evaluate whether or not the data support a particular model or group of models.

There are essentially two components that we must specify in the HB model: the likelihood (i.e., the data model) and all prior distributions for the unknown quantities (i.e., the parameter model). In some applications, one may also specify a probabilistic process model to account for “process error,” which describes additional variation not explained by measurement error and the mean model (e.g., in this case, the mean model is given by one of the scaling models). However, we do not explicitly separate process error from measurement error because the data do not facilitate separation of these two error terms; separation would be enabled by, for example, repeated measurements on at least a subset of individuals (Ogle & Barber 2008). Thus, we estimate the combined measurement and process variance and focus on the parameter model and its ability to explain the variation in the data. That is, each scaling model (Table 1) results in a different parameter model, but the likelihood part remains unchanged. The HB model gives the joint posterior distribution for all unknown quantities, which is proportional to the likelihood multiplied by the prior(s) (Gelm*an et a*l. 2004).

**Defining the likelihood**

We define the likelihood based on the following assumptions. For observation *i* in dataset *k* with *N_k_* observations, (*i* = 1, 2, 3, …, *N_k_*), without loss of generality, we let *r_i_* serve as the independent variable. A Berkson type model accounts for potential errors in *r_i_* (Dellaportas & Stephens 1995), and based on exploratory analyses, we assume that the errors are log-normally distributed:

$$\log\left(\rho_{i}\right)\sim N\left(\log\left(r_{i}\right),\sigma_{r}^{2}\right)$$

*ρ_i_* is the “true” or latent radius and$\sigma_{r}^{2}$is the observation or measurement variance. For a given dataset, we only allow the most flexible model or the “full” model (i.e., SPAM) to inform the latent radii values. Thus, all models utilize the same *ρ_i_* values, thereby eliminating potential differences between models that could be solely due to different estimates of the latent radii.

Next, we allow for the possibility that observation (and process) errors associated with the other variables (*l*, *A*, *M*) are correlated, and we also assume that each is log-normally distributed. On the log-scale, the multivariate normal likelihood for observation vector *i* is:

$$\left[\begin{matrix} \log(l_{i}) \\ \log(M_{i}) \\ \log(A_{i}) \end{matrix}\right]\sim N\left(\left[\begin{matrix} \alpha_{l,s(i)} & \beta_{l,s(i)} \\ \alpha_{M,s(i)} & \beta_{M,s(i)} \\ \alpha_{A,s(i)} & \beta_{A,s(i)} \end{matrix}\right]\left[\begin{matrix} 1 \\ \log(\rho_{i}) \end{matrix}\right],\Sigma\right)$$

The *α*’s are the normalizing constants, and the *β*’s are the scaling exponents for the relationships between *l*, *M*, or *A* (as per the subscripts) and the latent radius.  is a 3×3 covariance matrix. The subscript *s*(*i*) indicates “species *s* associated with observation *i*,” and thus Eqn. 2 represents the most flexible model (i.e., SPAM; see Dietze et al. 2008 for a similar approach). For all scaling models, we allow the α’s to differ between species. We may modify the model with respect to the *β*’s such that we may drop the *s*(*i*) subscript for models that assume that the exponents do not differ between species and we may also assume that the *β*’s take-on specific values (e.g., see the universal models; Table 1). Such modifications would reduce the complexity of the model by reducing the number of free parameters. Note also that the three different *β* ’s (i.e., *β_l_*, *β_M_*, and *β_A_*) relate to the predicted exponents in Table 1 associated with the *r* column. Together, Eqns. 1 and 2 define a component of the likelihood. The likelihood (of all data) is obtained by taking the product of all components across all observations by assuming conditional independence; i.e., each observed vector is assumed independent of the other observed vectors given the true values (i.e., the latent, predicted, or mean values).

Next we define the parameter model. Consider the most flexible model, as depicted by Eqn. 2. We employ a hierarchical prior that essentially treats species as random effects whereby we model species-specific parameters as coming from an overall population (or “global” distribution) that is defined by population-level parameters (or hyperparameters) (e.g., Cla*rk et a*l. 2005; Ogle & Barber 2008). For example, for variable *Y* (*Y* = *l*, *A*, or *M*) and species *s*:

$$\begin{matrix} \alpha_{Y,s}\sim N\left(\tilde{\alpha}_{Y},\sigma_{\alpha Y}^{2}\right) \\ \beta_{Y,s}\sim N\left(\tilde{\beta}_{Y},\sigma_{\beta Y}^{2}\right) \end{matrix}$$

$\tilde{\alpha}_{Y}$ and $\tilde{\beta}_{Y}$ are the overall, population-level intercepts and scaling exponents, respectively, for the relationship between variable *Y* and the latent radius (*ρ*). The variances, $\sigma_{\alpha Y}^{2}$ and $\sigma_{\beta Y}^{2}$, describe variability between species with respect to the species-specific intercepts and scaling exponents. If these variances are “small” then this implies that species have relatively “similar” parameter values, and the parameter values for under-represented species will be partially constrained by the values estimated for the well-represented species, resulting in “borrowing of strength” between species. If the variances are “large” then species potentially differ in their parameter values, and the under-represented species will not be informed by well-represented species, resulting in wide posterior interval estimates for the under-represented species’ parameters.

**Specifying Prior Distributions**

Finally, we conclude the HB model by specifying prior distributions for all remaining parameters, including all (co)variance terms (e.g., the σ^2^’s in Eqns. (1) and and Σ in Eqn. (2) and all hyperparameters (e.g., the $\tilde{\alpha}_{Y}$’s and $\tilde{\beta}_{Y}$’s in Eqn. ). Because repeated measurements were not available for *r*, which would help to inform the estimate of $\sigma_{r}^{2}$in Eqn. (1), we specified a relatively informative prior for $\sigma_{r}^{2}$ based on reports that the coefficient of variation (cv) of tree diameter measurement errors is generally less than 5% (Elzin*ga et a*l. 2005). Since *r* is modeled on the log-scale, *σ_r_* is approximately equal to the coefficient of variation (cv) on the regular, non-log scale for small cv (i.e., for cv < 0.5). The log-normal prior that we chose resulted in a prior mean or expected value of *σ_r_* equal to 0.021 and the prior probability that *σ_r_* exceeds 0.05 is equal to 0.054. We specified standard, non-informative (diffuse) priors for all other parameters, including: diffuse gamma densities for the remaining precisions terms (i.e., 1/σ^2^); a non-informative Wishart distribution for the precision matrix (i.e., Σ^–1^); and diffuse normal densities (mean of zero and large variances) for the $\tilde{\alpha}_{Y}$’s and $\tilde{\beta}_{Y}$’s (for a discussion of these “standard” priors, see Gelm*an et a*l. 2004).

Note that the above parameter model describes the SPAM model whereby $\beta_{l}$, $\beta_{M}$, and $\beta_{A}$in Eqns. and are equivalent to the scaling exponents *η*, **, and *λ*, respectively in Table 1. The parameter model was adjusted slightly to accommodate the other scaling models. For all models, the parameter model for the *α*’s, $\tilde{\alpha}_{Y}$’s, and $\sigma_{\alpha Y}^{2}$ remained unchanged, but we may modify the parts related to the scaling exponents (i.e., the *β*’s, $\tilde{\beta}_{Y}$’s, and $\sigma_{\beta Y}^{2}$). For example, for the universal scaling models, we do not have a stochastic parameter model for the scaling exponents because the *β*’s are fixed according to the predicted values in Table 1. For the constrained exponents models (e.g., PES, Table 1), the parameter model for the *β*’s is reduced to a model for two exponents. That is, we modeled $\beta_{l}$and$\beta_{A}$ according to Eqn. , and based on the relationships among the exponents and the parameters *a* and *b* in Table 1, $\beta_{M}$=$\beta_{l}$+ 2, *a* = 1/$\beta_{A}$, and *b* =$\beta_{l}$/$\beta_{A}$.

**Mass-based analysis**

As indicated by Eqns. (1) and (2), we chose radius (*r*) to serve as the independent variable, but we could have chosen one of the other variables such as mass (*M*) or length (*l*). The choice of which variable to use as the independent variable should not matter because (1) we account for uncertainty (or error) in all variables and all are linked via their latent (mean or predicted) values through the scaling models, and (2) we assume that the errors are additive on the log scale. To verify that our choice of *r* as the independent variable did not bias our results, we performed a parallel analysis with *M* as the independent variable. The results from this analysis did not differ qualitatively from those with radius as the independent variable, and are presented in Figure S1.

**Implementation**

We used Markov chain Monte Carlo (MCMC) methods to approximate the joint posterior distribution associated with the above likelihood and parameter models. We implemented the models in WinBUGS (Lu*nn et a*l. 2000), a general-purpose statistical software package for conducting Bayesian analyses. The procedure for obtaining the posterior distributions associated with each of the candidate models was applied to all of the plant and leaf datasets as follows. For a given dataset, all models were simultaneously implemented because they shared the same latent radii. In all cases, we ran three parallel MCMC chains; starting values for each chain were based on initial runs where we specified widely dispersed values for global parameters and precision terms and used WinBUGS to generate starting values for other parameters based on the priors. All chains converged by iteration 5,000, and we discarded these initial 5,000 samples as the burn-in period. Convergence was confirmed using the Brooks-Gelman-Rubin convergence statistic (Brooks & Gelman 1998). The remaining (converged) MCMC samples were thinned to reduce or eliminate within chain autocorrelation, thereby yielding an independent or nearly independent sample from the joint posterior. For the MCMC simulations that used the Cannell data, 220,000 iterations were run per chain and the chains were thinned every 100, yielding a posterior sample size of 6,450. For the Sonoran and leaf datasets, 165,000 iterations were run per chain, chains were thinned every 50, and the total sample size was 9,600.

We compared the abilities of the different scaling models to fit the observed data by computing the posterior predictive loss (*D*) for each model/dependent variable combination (Gelfand & Ghosh 1998). *D* provides an index of a model’s predictive ability by comparing observed data (e.g., observations of *l*, *M*, or *A*) to “replicated data” (Gelm*an et a*l. 2004) that are generated from the same sampling distribution (i.e., from Eqn. (2)). The computation of *D* is based on a squared-error loss function that penalizes for departure from the observed data (a measure of goodness-of-fit) and for model “smoothness” (a measure of model complexity) (Gelfand & Ghosh 1998). *D* can be partitioned into these two components, but we simply report the overall value of *D* for each model, variable (*l*, *M*, and *A*), and combinations of variables (e.g., *l* and *M* or *l*, *M*, and *A*). The *D* values were computed for the log-scale variables. The model with a smaller *D* is preferred over models with larger *D* values.

We also evaluated model goodness-of-fit by creating observed vs. predicted plots for each measured plant characteristic. The predicted values that we used were the posterior means and 95% Bayesian credible intervals (BCIs) for replicated data (Gelm*an et a*l. 2004). We also evaluated whether or not each model was, or was not, consistent with the observed data by determining if the hypothesized scaling coefficients for each model (Table 1) were contained within the 95% BCIs for the species-specific and/or population-level scaling exponents associated with the most flexible model (i.e., as described above; also the same as the SPAM model, Table 1).

We applied the HB models and ran the MCMC simulations separately for each dataset. In theory, one could combine the Sonoran and Cannell datasets into one analysis because both provide data representative of whole plants. However, the methods used to obtain the measurements in each dataset differed, each contained completely different groups of species, and each represents different biological scales. That is, the Cannell dataset represents the “average tree” based on averaging information across entire stands while the Sonoran dataset provides raw data on individual plants. Thus, we elected to analyze the datasets separately.

**Literature Cited**

Brooks S.P. & Gelman A. (1998). General methods for monitoring convergence of iterative simulations. *J. Comput. Graph. Stat.*, 7, 434-455.

Carlin B.P., Clark J.S. & Gelfand A.E. (2006). Elements of hierarchical Bayesian inference. In: *Hierarchical Modelling for the Environmental Sciences: Statistical Methods and Applications*. Oxford University Press New York, pp. 3-24.

Clark J.S., Ferraz G.A., Oguge N., Hays H. & DiCostanzo J. (2005). Hierarchical Bayes for structured, variable populations: From recapture data to life-history prediction. *Ecology*, 86, 2232-2244.

Dellaportas P. & Stephens D.A. (1995). Bayesian analysis of errors-in-variables regression models. *Biometrics*, 51, 1085-1095.

Elzinga C., Shearer R.C. & Elzinga G. (2005). Observer variation in tree diameter measurements. *West. J. Appl. For.*, 20, 134-137.

Gelfand A.E. & Ghosh S.K. (1998). Model choice: a minimum posterior predictive loss approach. *Biometrika*, 85, 1-11.

Gelman A., Carlin J.B., Stern H.S. & Rubin D.B. (2004). *Bayesian Data Analysis*. Chapman and Hall/CRC Press, Boca Raton.

Lunn D.J., Thomas A., Best N. & Spiegelhalter D. (2000). WinBUGS - A Bayesian modelling framework: Concepts, structure, and extensibility. *Statistics and Computing*, 10, 325-337.

Ogle K. & Barber J.J. (2008). Bayesian data-model integration in plant physiological and ecosystem ecology. *Progress in Botany*, 69, 281-311.

**Figure S1:** Posterior distributions for the global exponents in the SPAM model. The dashed vertical lines represent exponent values predicted by the universal models (Table 1). None of the universal models enjoys strong support across all allometries or all datasets. Note that the elastic similarity model makes the same predictions as WBE for the scaling of mass and length. In addition, stress and elastic similarity models do not make predictions for the scaling of surface area.

| **Model** | **Dataset** | **sd(*l*)** | **sd(*M*)** | **sd(*A*)** | **cor(*l*,*A*)** | **cor(*l*,*M*)** | **cor(*M*,*A*)** |
| --- | --- | --- | --- | --- | --- | --- | --- |
| ELASTIC | Cannell | 0.112 | 0.129 | NA | NA | 0.035 | NA |
| STRESS | Cannell | 0.186 | 0.167 | NA | NA | -0.022 | NA |
| GEOM | Cannell | 0.123 | 0.242 | 0.210 | **0.475** | **0.134** | **0.686** |
| WBE | Cannell | 0.112 | 0.185 | 0.210 | 0.034 | **-0.307** | **0.623** |
| PES | Cannell | 0.115 | 0.176 | 0.169 | -0.031 | **-0.312** | **0.515** |
| SPAM | Cannell | 0.107 | 0.128 | 0.144 | **0.209** | **-0.199** | **0.290** |
| ELASTIC | Sonoran | 0.199 | 0.344 | NA | NA | **0.475** | NA |
| STRESS | Sonoran | 0.216 | 0.326 | NA | NA | **0.428** | NA |
| GEOM | Sonoran | 0.225 | 0.408 | NA | NA | **0.679** | NA |
| WBE | Sonoran | 0.199 | 0.344 | NA | NA | **0.476** | NA |
| PES | Sonoran | 0.182 | 0.335 | NA | NA | **0.432** | NA |
| SPAM | Sonoran | 0.182 | 0.298 | NA | NA | **0.535** | NA |
| ELASTIC | Leaves | 0.158 | 0.182 | NA | NA | **0.332** | NA |
| STRESS | Leaves | 0.184 | 0.180 | NA | NA | **0.408** | NA |
| GEOM | Leaves | 0.119 | 0.204 | 0.199 | **0.339** | **0.809** | **0.453** |
| WBE | Leaves | 0.159 | 0.183 | 0.199 | **0.334** | **0.781** | **0.658** |
| PES | Leaves | 0.077 | 0.181 | 0.113 | -0.019 | **0.482** | **0.590** |
| SPAM | Leaves | 0.072 | 0.103 | 0.105 | **0.435** | **0.656** | **0.570** |

**Table S4.** Posterior means for terms associated with the covariance matrix Σ in Eqn. (2); sd = standard deviation; cor = correlation coefficient, e.g., cor(*x*,*y*) is the correlation between trait *x* and *y* after accounting for covariation explained by the scaling model; bolded numbers indicated correlations that are significantly different from zero (i.e., their 95% BCIs did not contain zero); NA = not applicable. The correlation coefficients were particularly high for the Sonoran and leaf datasets, and the magnitude of the correlations did not vary systematically by scaling model.
