# Supplementary material for: Evaluating scaling models in biology using hierarchical Bayesian approaches
Source: Ecol Lett. 2009 Jul;12(7):641–51. doi: 10.1111/j.1461-0248.2009.01316.x (PMC2730548; doi:10.1111/j.1461-0248.2009.01316.x)
Supplement: Appendix S3 — WinBUGS code. [file ele0012-0641-SD2.doc]

**SUPPORTING INFORMATION**

**Appendix S3**

**Methods**

WinBUGS code used for the Cannell and leaf datasets (both contained data on all four traits: *r*, *l*, *M*, and *A*). The WinBUGS code used for the Sonoran data is a slightly modified (or simplified) version of the following code because the Sonora data only included data on three traits (*r*, *l*, and *M*).

**model**

{

# Loop through each observation i in the dataset:

for(i in 1:N){

# Create data matrix by repeating observed data 6 times, for each of the six models.

# The data are read-in in a file that contains the variables LogLength = log(l), LogMass =

# log(M), and logArea = log(A). Put all data in the data vector Y, which is equivalent to the

# observation vector for [log(l) log(M) log(A)] in eqn (2) in the main text.

for(m in 1:6){

Y[i,m,1] <- LogLength[i]

Y[i,m,2] <- LogMass[i]

Y[i,m,3] <- LogArea[i]

}

# The **likelihood** (sampling distribution for loglength, logmass, logarea) is a

# multivariate normal distribution with mean mu and precision matrix Omega.

# Note, the text refers to the covariance matrix, Sigma, but WinBUGS parameterizes

# the normal distribution in terms of a precision matrix.The data are Y[,,1] = loglength;

# Y[,,2] = logmass; Y[,,3] = logarea. Allow the mean and precision to vary by model m.

# If models 1 (elastic) or 2 (stress), only use LogLength & LogArea, Y[i,m,1:2] and use

# the mean mu12 and precision matrix Omega12; for the other models, use all three

# variables, Y[i,m,1:3], and use the mean mu and the precision matrix Omega

for(m in 1:2){

# Multivariate normal likelihood for first 2 scaling models:

Y[i,m,1:2] ~ dmnorm(mu12[i,m,1:2], Omega12[m,1:2,1:2])

# Define **replicated** **data**, Yrep12, for each model

Yrep12[i,m,1:2] ~ dmnorm(mu12[i,m,1:2], Omega12[m,1:2,1:2])

for(k in 1:2){

# Compute squared difference (or squared error) for **posterior**

# **predictive** **loss** calculation:

sqdiff12[i,m,k] <- pow(Yrep12[i,m,k] - Y[i,m,k],2)

# Replicated data on regular, non-log scale

eYrep12[i,m,k] <- exp(Yrep12[i,m,k])

}

}

# Now do the remaining models that involve area:

for(m in 3:6){

# Multivariate normal likelihood for each scaling model:

Y[i,m,1:3] ~ dmnorm(mu[i,m-2,1:3], Omega[m-2,1:3,1:3])

# mu & Omega are indexed by m-2 so that they have 1,..,4 "elements" for these 4 models

# Define replicated data, Yrep, for each model

Yrep[i,m-2,1:3] ~ dmnorm(mu[i,m-2,1:3], Omega[m-2,1:3,1:3])

for(k in 1:3){

# Compute squared difference (or squared error) for posterior

# predictive loss calculation:

sqdiff[i,m-2,k] <- pow(Yrep[i,m-2,k] - Y[i,m,k],2)

# Replicated data on regular, non-log scale

eYrep[i,m-2,k] <- exp(Yrep[i,m-2,k])

}

}

# Define the **mean** **vector** (i.e., scaling model that relates the true or latent variables).

# alpha is the species-specific normalizing constant, and beta is the species-specific

# scaling exponent.

for(m in 1:2){

for(k in 1:2){

mu12[i,m,k] <- alpha[SP[i],m,k] + beta[SP[i],m,k]*Lrho[i,m]

}

}

for(m in 3:6){

for(k in 1:3){

mu[i,m-2,k] <- alpha[SP[i],m,k] + beta[SP[i],m,k]*Lrho[i,m]

}

}

# **Berkson model for "true" or latent log diameter** (Lrho). Note, the data are in terms of

# diameter, and the use of diameter vs radius will not affect the scaling exponents.

# Lrho varies about measured LogDiameter, and tauD is the precision (1/variance) that

# describes measurement errror. The Berkson model is for "LatentRho", which is used by

# and updated by SPAM-data (hence, Lrho[6,i] = LatentRho[i]). But, feedback from the other

# data-model combinations is severed by using the cut function. Thus, the SPAM model

# "drives" the estimates of the latent diameter (radius), and the same latent diameters (radii)

# are then used in the other models.

LatentRho[i] ~ dnorm(LogDiameter[i], tauD)

for(m in 1:5){

# For use with models 1-5:

Lrho[i,m] <- cut(LatentRho[i])

}

# For use with model 6 (SPAM) -- no cutting:

Lrho[i,6] <- LatentRho[i]

} # close observation (i) loop

# Compute **posterior predictive loss** for each trait & model, and combined for each model.

# For models 1 and 2 (don't involve area):

for(m in 1:2){

for(k in 1:2){

# Sum of squared diff for each model and trait variable (sum across observations):

Dsum12[m,k] <- sum(sqdiff12[,m,k])

}

# Sum of squared diff for each model; sum across observations and traits (for length & mass)

DsumM12[m] <- sum(Dsum12[m,])

}

# For models 3-6 (include area):

for(m in 3:6){

for(k in 1:3){

# Sum of squared diff for each model and trait variable (sum across observations):

Dsum[m-2,k] <- sum(sqdiff[,m-2,k])

}

# Sum of squared diff for each model; sum across observations and across common traits

# (i.e., length & mass)

DsumM[m-2] <- sum(Dsum[m-2,1:2])

# Sum of squared diff for each model; sum across obs and across all traits

# (i.e., length, mass, and area)

DsumM.all[m-2] <- sum(Dsum[m-2,])

}

# Model specification for **species-level scaling parameters, based on the scaling model.**

**#** Scaling exponents are allowed to vary (independently) by model

for(j in 1:Nsp){

# Scaling exponent (beta) model:

for(m in 1:6){

for(k in 1:3){

# Set scaling exponent to correct value according to the model (m) and

# trait (m) of interest:

beta[j,m,k] <- universal.part[j,m,k] + pes.part[j,m,k] + spam.part[j,m,k]

# Universal model specification (read in fixed values for associated constant.beta).

# universal.part = 0 if m > 4.5 (i.e., for m = 5 & 6); otherwise, equal to constant.beta

universal.part[j,m,k] <- step(4.5-m)*constant.beta[m,k]

# PES model specification, pes.part = beta.pes when m = 5; otherwise zero.

pes.part[j,m,k] <- equals(5,m)*beta.pes[j,k]

# SPAM model specification; spam.part = beta.spam when m=6, otherwise 0.

spam.part[j,m,k] <- equals(6,m)*beta.spam[j,k]

}

}

# **Hierarchical** **priors** for species-specific exponents in the PES and SPAM models:

for(k in 1:3){

beta.spam[j,k] ~ dnorm(mu.beta.spam[k],tau.beta.spam[k])

}

beta.pes[j,1] ~ dnorm(mu.beta.pes[1],tau.beta.pes[1])

beta.pes[j,2] <- beta.pes[j,1] + 2

beta.pes[j,3] ~ dnorm(mu.beta.pes[3],tau.beta.pes[3])

# Compute PES parameters a and b in Table 1:

a.pes[j] <- 1/beta.pes[j,3]

b.pes[j] <- beta.pes[j,1]/beta.pes[j,3]

# Hierarchical priors for species-specific normalizing constants, for all models:

# For the first two models, only define the hierarchical priors for the l vs. r and

# M vs. r exponents:

for(m in 1:2){

for(k in 1:2){

alpha[j,m,k] ~ dnorm(mu.alpha[m,k], tau.alpha[m,k])

}

# Since no area predictions for models 1 and 2, just fill in area-scaling parameters

# with zeros, which simply serve as "place holders" and do not feedback to model.

alpha[j,m,3] <-0

}

# For the remaining models, define hierarchical priors for all three scaling exponents:

for(m in 3:6){

for(k in 1:3){

alpha[j,m,k] ~ dnorm(mu.alpha[m,k], tau.alpha[m,k])

}

}

} # close species (j) loop

# Hyperpriors for global parameters and priors for variance/precision terms:

# First define for the first 2 universal models that don't involve area:

for(m in 1:2){

Omega12[m,1:2,1:2] ~ dwish(R12[1:2,1:2], 2)

Sigma12[m,1:2,1:2] <- inverse(Omega12[m,1:2,1:2])

# Compute correlation between loglength & logmass errors:

rho12[m] <- Sigma12[m,1,2]/sqrt(Sigma12[m,1,1]*Sigma12[m,2,2])

for(k in 1:2){

# Prior for global normalizing constants and associated precisions

mu.alpha[m,k] ~ dnorm(0,0.00001)

tau.alpha[m,k] ~ dgamma(0.01,0.001)

# Compute standard deviation

sig.alpha[m,k] <- sqrt(1/tau.alpha[m,k])

}

# Place holders for first two models that don't involve area (the 3rd trait)

mu.alpha[m,3] <- 0

tau.alpha[m,3] <- 1

sig.alpha[m,3] <- 1

}

# Then define for the remaining four models that involve area:

for(m in 3:6){

Omega[m-2,1:3,1:3] ~ dwish(R[1:3,1:3], 3)

Sigma[m-2,1:3,1:3] <- inverse(Omega[m-2,1:3,1:3])

# Compute correlations between loglength, logmass, and logarea errors:

rho[m-2,1] <- Sigma[m-2,1,2]/sqrt(Sigma[m-2,1,1]*Sigma[m-2,2,2]) # length/mass

rho[m-2,2] <- Sigma[m-2,1,3]/sqrt(Sigma[m-2,1,1]*Sigma[m-2,3,3]) # length/area

rho[m-2,3] <- Sigma[m-2,3,2]/sqrt(Sigma[m-2,3,3]*Sigma[m-2,2,2]) # mass/area

# Prior for global normalizing constants and associated precisions

for(k in 1:3){

mu.alpha[m,k] ~ dnorm(0,0.00001)

tau.alpha[m,k] ~ dgamma(0.01,0.001)

# Compute standard deviation

sig.alpha[m,k] <- sqrt(1/tau.alpha[m,k])

}

}

# Priors for global scaling exponent parametes for PES and SPAM models:

mu.beta.pes[1] ~ dnorm(0, 0.00001)

mu.beta.pes[2] <- mu.beta.pes[1] + 2

mu.beta.pes[3] ~ dnorm(0, 0.00001)

mu.a.pes <- 1/mu.beta.pes[3]

mu.b.pes <- mu.beta.pes[1]/mu.beta.pes[3]

tau.beta.pes[1] ~ dgamma(0.01,0.001)

tau.beta.pes[3] ~ dgamma(0.01,0.001)

# Since beta2 = beta1 + 2, var(beta2) = var(beta1+2) = var(beta1);

# thus, precision(beta1) = precision(beta2):

tau.beta.pes[2] <- tau.beta.pes[1]

for(k in 1:3){

sig.beta.pes[k] <- sqrt(1/tau.beta.pes[k])

mu.beta.spam[k] ~ dnorm(0,0.00001)

tau.beta.spam[k] ~ dgamma(0.01,0.001)

sig.beta.spam[k] <- sqrt(1/tau.beta.spam[k])

}

# Prior for precision of logRadius measurement error. Use fairly informative prior

# based on, for example, Elzinga et al. (2005), Observer variation in tree diameter

# measurements. Western Journal of Applied Forestry, 20:134-137, which says

# "Measurement error rates of 5% of tree diameter or greater may be expected

# in dbh measurements on as many as 5% of measured trees." Thus, choose

# lognormal prior for sigD that gives E(sigD) = 0.021 and P(sigD > 0.05) = 0.054;

# Note, sigD describes measurement error on the log-scale and thus can be

# interpretted as the "multiplicative" or "percent" error rate.

sigD ~ dlnorm(-4.135, 2)

tauD <- pow(sigD, -2)

}

# Read in sample size and R matrix (example for Cannell data):

list(N=**256**, Nsp=**14**,R12=structure(.Data=c(1,0,0,1),.Dim=c(2,2)), R=structure(.Data=c(1,0,0,0,1,0,0,0,1),.Dim=c(3,3)),

constant.beta=structure(.Data=c(

0.666667, 2.666667, **0**,

0.5, 2.5, **0**,

1, 3, 2,

0.666667, 2.666667, 2,

**0,0,0,**

**0,0,0**),.Dim=c(6,3)))

# Note: just fill-in unused elements of constant.aphla with zeros (**0**).

# Example of the trait data file (e.g., rectangular array format) based on

# the Cannell dataset

SP[] LogArea[] LogMass[] LogLength[] LogDiameter[]

1 4.647250147 5.699649829 4.372912003 2.551887354

1 4.379508906 5.286348221 4.238046103 2.461989937

1 4.208543283 5.324708095 4.320146286 2.430819006

.....

14 4.68613278 6.128089617 4.437750563 2.685031953

14 4.378131615 5.840529613 4.525044807 2.547205475

14 4.359642807 5.835858338 4.536558443 2.527111664

14 4.271375714 5.721624822 4.509202522 2.515391621

END
